# Supplementary material for: Harvesting energy from sun, outer space, and soil
Source: Sci Rep. 2020 Dec 1;10:20903. doi: 10.1038/s41598-020-77900-7 (PMC7708839; doi:10.1038/s41598-020-77900-7)
Supplement: Supplementary file 1 — Supplementary material 1 [file 41598_2020_77900_MOESM1_ESM.pdf]

Supplementary Information

Harvesting Energy from Sun, Outer Space, and Soil

Yanpei Tian[1], Xiaojie Liu[1], Fangqi Chen[1], Yi Zheng[1,2*]

[1]Department of Mechanical and Industrial Engineering, Northeastern University, Boston, MA, USA.

[2]Department of Electrical and Computer Engineering, Northeastern University, Boston, MA, USA.

[*] e-mail: y.zheng@northeastern.edu

Theoretical Model:

A theoretical model is developed to characterize the performance of the TEG based system for the outdoor performance and also to get the extrapolated performance under different environmental parameters and various weather conditions. Here, it is assumed that there is no heat loss from the PS insulation box clothed with the highly reflective silver mylar films. In this model, the energy balance of the TEG device for the daytime is:

$$P_{net}^{heating} = P_s(T_{Black}) - P_r(T_{Black}) - P_{nr}(T_{Air}, T_{Black}) + P_a(T_{Air}) - P_c(T_{Black}, T_{Soil}) \quad (1)$$

where the P_s is the absorbed solar intensity of the black absorber. P_r stands for the radiative cooling power of the black absorber. P_{nr} is the non-radiative power between the black absorber and the ambient air. P_a represents the incident thermal radiative power from the ambient. P_c means the heat conduction flux between the TEG device and the soil. Here, T_{Black} is the temperature of the black absorber/emitter's top side. T_{Air} stands for the ambient temperature, and T_{Soil} represents the soil temperature. The radiative power exchange between the space and the ambient can be neglected as it plays a non-dominant role in analyzing the net heating and cooling power of the entire energy harvesting system during the daytime and nighttime.

The energy balance at night is:

$$P_{net}^{cooling} = P_r(T_{Black}, T_{Space}) - P_{nr}(T_{Air}, T_{Black}) - P_a(T_{Air}) - P_c(T_{Black}, T_{Soil}) \quad (2)$$

The heat generation and absorption due to the Seebeck effect and Joule heating in the TEG module are neglected due to the power conversion efficiency of the TEG module is below 0.5% even at daytime for the outdoor testing. The solar intensity of the absorber is given by:

$$P_r(T_{Black}) = I_{Solar}(t) \cdot \epsilon(T_{Black}) \quad (3)$$

where, I_{Solar} is the solar intensity variations measured by the weather station during the outdoor testing. $\epsilon(T_{Black})$ is the overall temperature-dependent solar absorptance of the black absorber and its variations with the temperature are neglected and the measured spectrum at room temperature (25°C) is taken into analysis. P_r can be determined as follows:

$$P_r(T_{Black}) = \int_0^{\infty} d\lambda I_{BB}(T_{Black}, \lambda) \epsilon(\lambda, \theta, \phi, T_{Black}) \quad (4)$$

where $I_{BB}(T_{Black}, \lambda) = 2hc^2\lambda^{-5}\exp(hc / \lambda k_B T_{Black} - 1)^{-1}$ defines the spectral radiance of blackbody at a certain temperature. Here, h is the Planck's constant, k_B is the Boltzmann constant, and λ is the wavelength. $\epsilon(\lambda, \theta, \phi, T_{Black}) = 1/\pi \int_0^{2\pi} d\phi \int_0^{\pi/2} \epsilon_\lambda \cos\theta \sin\theta d\theta$ is the temperature-dependent emissivity of the black absorber. Here, the emissivity measured at room temperature (25°C) is taken into the simulation. θ and ϕ are the azimuthal and latitudinal angles of the sun on the specific days of the outdoor testing. The non-radiative heat transfer between the absorber/emitter and the ambient air can be expressed as:

$$P_{nr}(T_{Air},T_{Black}) = h_{nr}(T_{Air} - T_{Black}) \tag{5}$$

where, h_{nr} is the non-radiative heat transfer coefficient. The absorbed power of the incident thermal radiation from the atmosphere $P_a(T_{Air})$ is given by:

$$P_a(T_{Air}) = \int_0^{\infty} d\lambda I_{BB}(T_{Air},\lambda)\epsilon(\lambda,\theta,\phi,T_{Black})\epsilon(\lambda,\theta,\phi) \tag{6}$$

where, the absorptivity of the atmosphere, $\epsilon(\lambda,\theta,\phi)$ is given by $1-\tau(\lambda,\theta,\phi)$. Here $\tau(\lambda,\theta,\phi)$ is the transmittance value of the atmosphere obtained from MODTRAN 4. P_c is given by:

$$P_c(T_{Black},T_{Soil}) = R_{TEG}(T_{Black} - T_{Soil}) \tag{7}$$

where R_{TEG} represents the thermal resistance of the TEG module.

The time-dependent temperature variations of the black absorber's topside can be obtained by solving the following equation:

$$P_{net} = \sum_i C_i \frac{dT_i}{dt} \tag{8}$$

where, the heat capacitance, C_i, represents heat capacitance of the black paint layer, the copper sheet, the thin layer of thermal compound paste, the TEG module, and the aluminum heat sink (details of these value are provided in Table S1). These elements have various temperatures, and their temperatures will evolve accordingly.

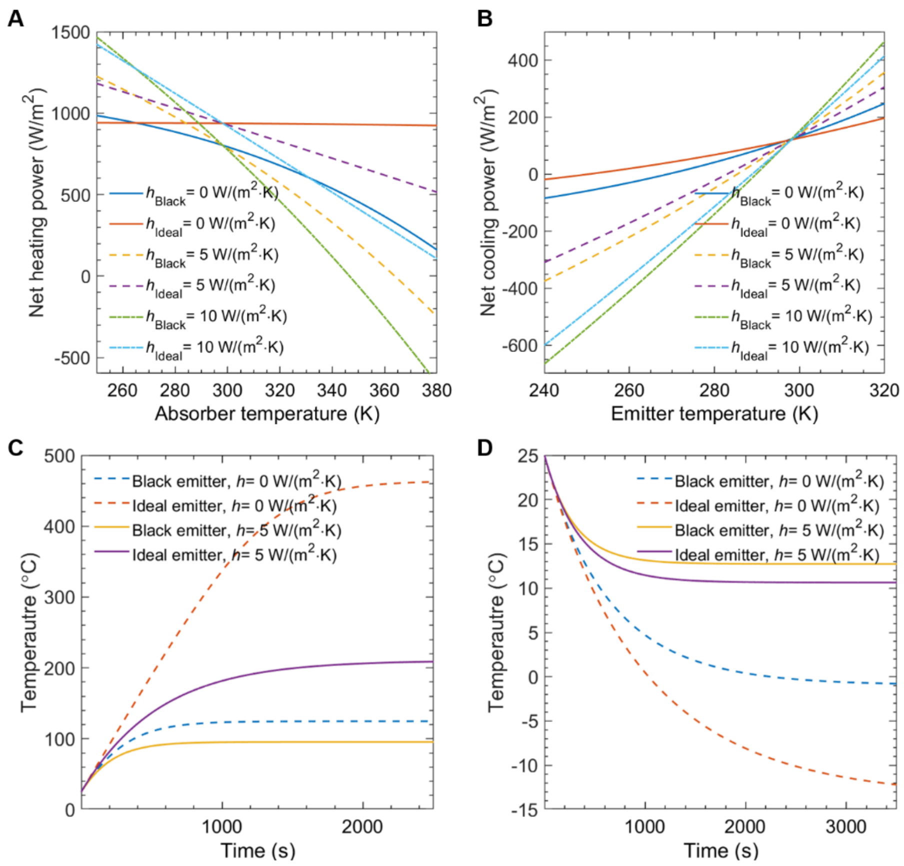

Supplementary Figure 1. The net heating (**A**) and cooling (**B**) power of the black absorber/emitter at different weather conditions (h = 0, 5, and 10 W/m^2 • K) as a function of ideal or black absorber/emitter's temperature. The transient temperature of the ideal or black absorber/emitter when it is heated by the sunlight (**C**) or cooled by radiative cooling (**D**) at different weather conditions (h = 0 and 5 W/m^2 • K) as a function of time. The ambient is set to be constant at 298 K, the atmospheric transmittance is taken from MODTRAN 4, and the AM 1.5 data is used as the solar irradiance. The backside of the absorber/emitter is insulated.

For the simulation in Supplementary Figure 1, it is supposed that the backside of the absorber/emitter is well insulated, and it has no connection with the TEG module and the soil. Three different scenarios are different from the one simulated in Figure 4 (main text). The first one is in a vacuum (h_{nr} = 0), the second one is that the absorber/emitter stays open to the air but with a relatively mild convection environment (h_{nr} = 5 W/m^2 K), and the third represent the situation that the absorber/emitter is under relatively strong convection environment (h_{nr} = 5 W/m^2 K). The data of the incident solar intensity is taken from AM 1.5, and the absorber is faced normal to the sun. The absorptance of the absorber/emitter is angular- and polarization-independent. The transmittance spectrum of the air is taken from MODTRAN 4.

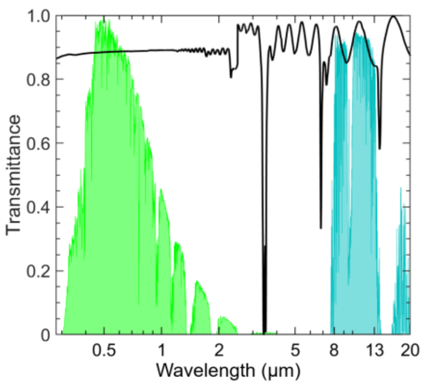

Supplementary Figure 2. The transmittance spectrum of the 12.7 μm LDPE film that is highly transparent form 0.3 μm to 20 μm.

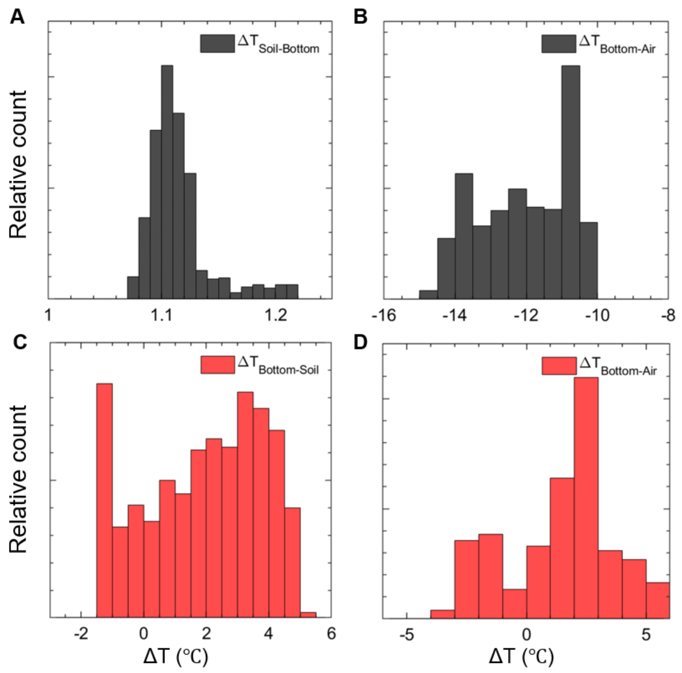

Supplementary Figure 3. Histogram of the temperature difference between the soil and the bottom side of the TEG module (**A**) and between the bottom side of the TEG side and the ambient air (**B**) during the nighttime and the daytime (**C**) and (**D**), respectively.

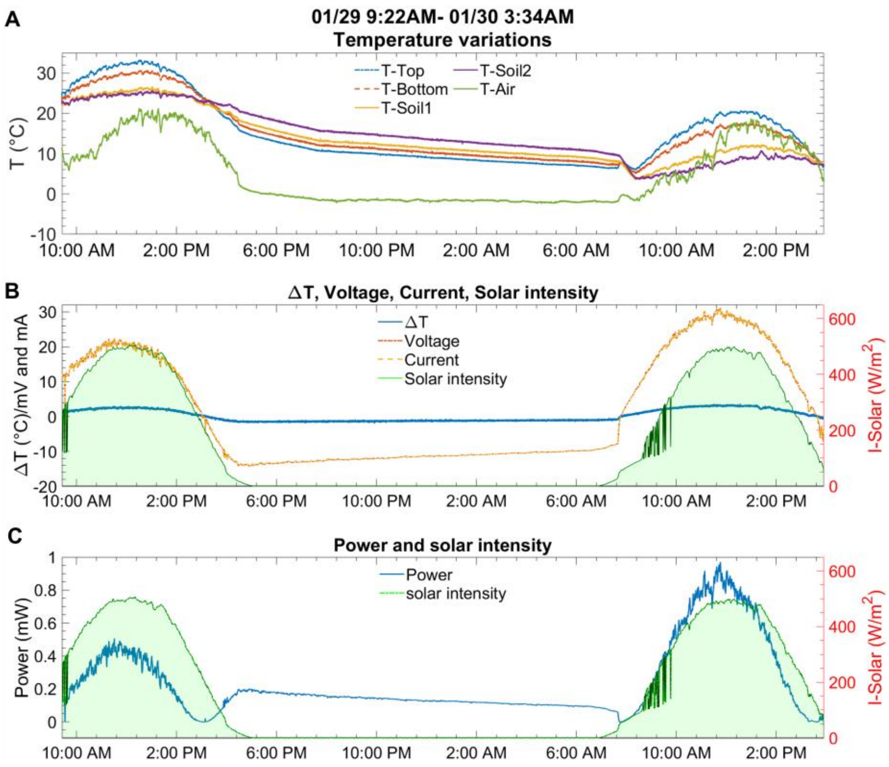

Supplementary Figure 4. (A) Temperature variations of the top and bottom surfaces of the TEG module, the soil, and ambient air from January 29, 2020, to January 30, 2020 (over 24-hour outdoor measurement). **(B)** The temperature difference between the top and bottom surfaces of the TEG module and its voltage and current output. **(C)** The output power of the TEG module displaying with the solar intensity.

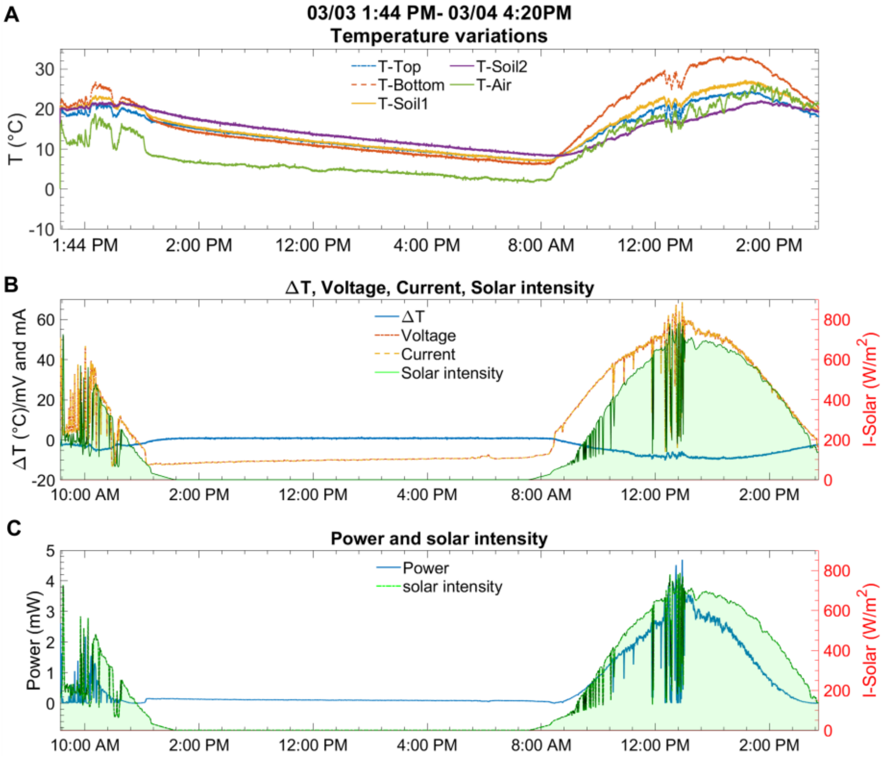

Supplementary Figure 5. (A) Temperature variations of the top and bottom surfaces of the TEG module, the soil, and ambient air from March 03, 2020, to March 04, 2020 (over 24-hour outdoor measurement). **(B)** The temperature difference between the top and bottom surfaces of the TEG module and its voltage and current output. **(C)** The output power of the TEG module displaying with the solar intensity.

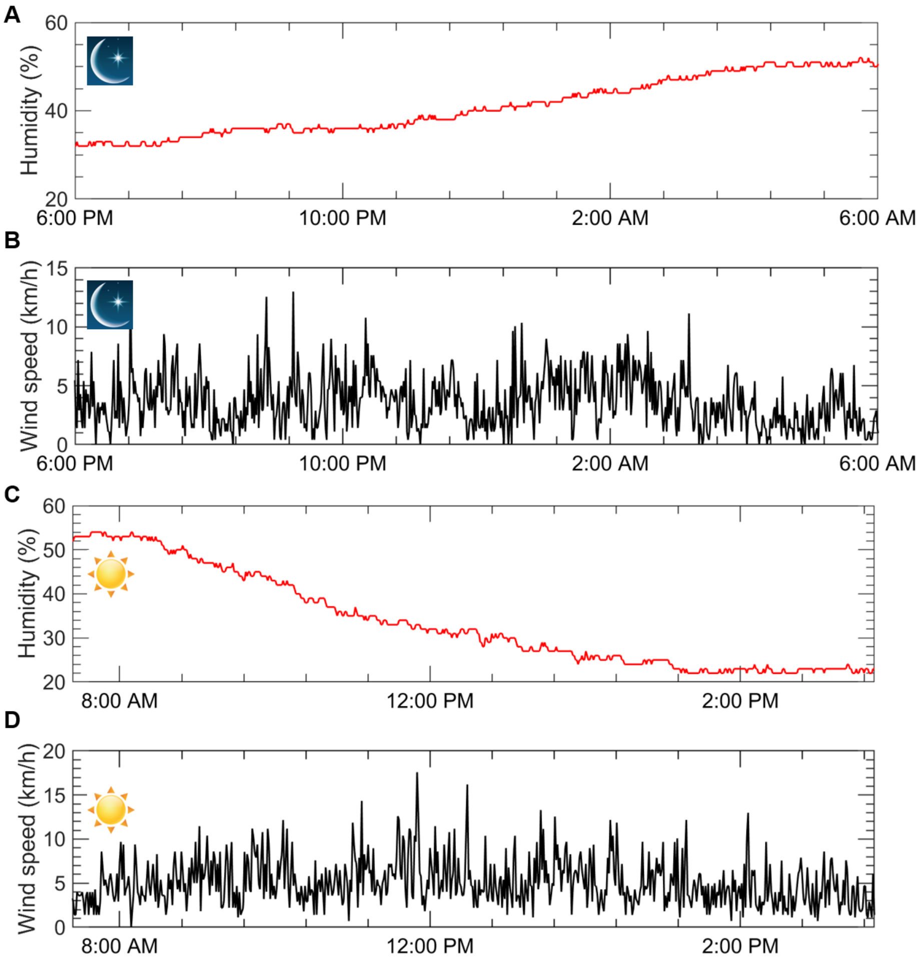

Supplementary Figure 6. (**A**) Relative humidity and (**B**) wind speed over the outdoor experiment for the nighttime (January 29 to January 30) and the daytime (**C**) and (**D**) (March 4), respectively.

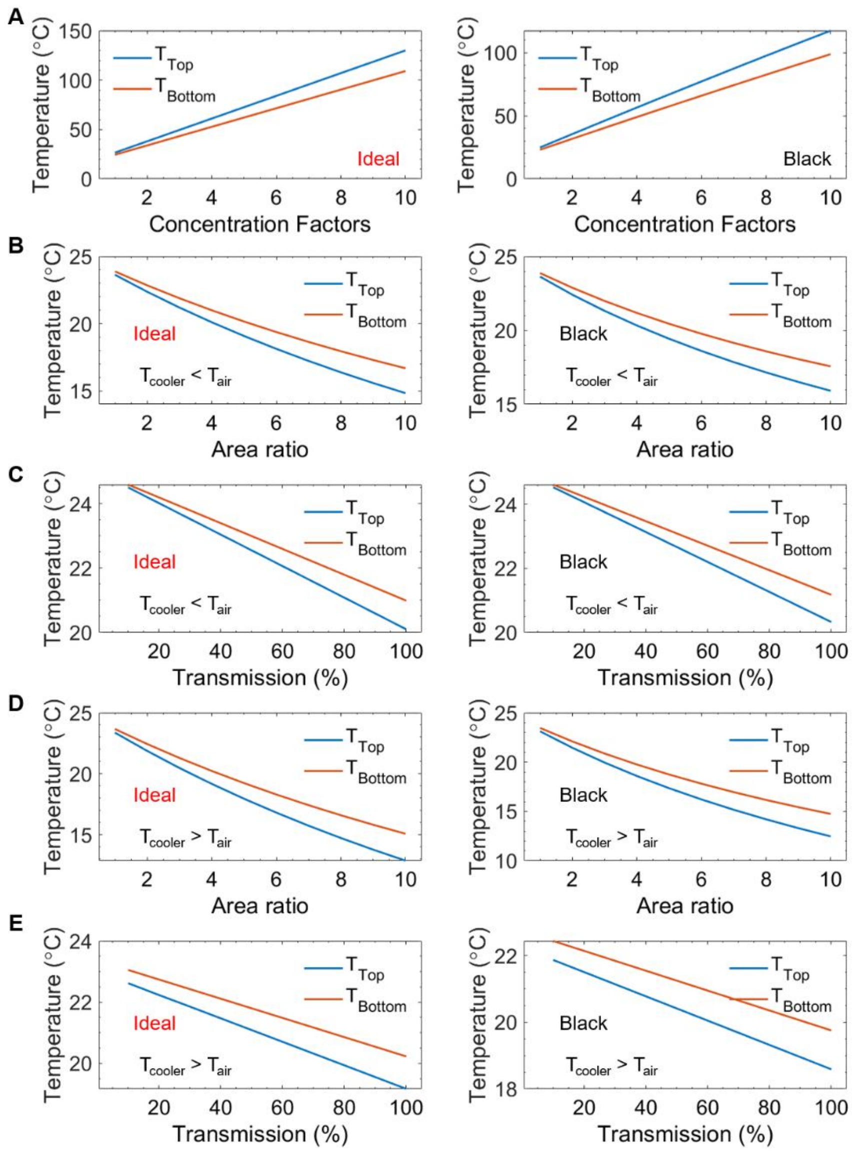

Supplementary Figure 7. The temperature of the TEG module's top and bottom sides under different concentration factors for the ideal and black absorber (**A**) when the absorber's area is the same as the TEG working surface. The ideal/black cooler's stagnation temperature of the top and bottom side as a function of the area ratio of the radiative cooler over the TEG's working surface (**B**) and under different atmospheric transmission (**C**) when the temperature of the radiative cooler drops below the ambient. (**D**) and (**E**) show the case when the temperature of the radiative cooler is above the ambient.

Table S1. The physical properties of different components of the TEG based energy harvesting system

Name	Thickness (*mm*)	Density (kg/m^3)	Specific heat capacity (*J/K·kg*)	Thermal conductivity (*W/m·K*)
Black 3.0 paint	0.1	1,180	1,466	2
Copper sheet	0.8	8,960	385	385
Top thermal paste	0.5	2,100	400	8
TEG module	3.4	4,136	120	1.6
Bottom thermal paste	0.5	2,100	400	8
Al heat sink	1.1	2,700	900	205
